# Establishment of Silane/GO Multistage Hybrid Interface Layer to Improve Interfacial and Mechanical Properties of Carbon Fiber Reinforced Poly (phthalazinone ether ketone) Thermoplastic Composites

**DOI:** 10.3390/ma15010206

**Published:** 2021-12-28

**Authors:** Shan Cheng, Nan Li, Yuxi Pan, Bing Wang, Haoyue Hao, Fangyuan Hu, Cheng Liu, Yousi Chen, Xigao Jian

**Affiliations:** 1 State Key Laboratory of Fine Chemicals, Department of Polymer Science and Materials, School of Chemical Engineering, Dalian University of Technology, Dalian 116024, China; 15610331427m@sina.cn (S.C.); pan.yuxi@163.com (Y.P.); 17864301035@163.com (B.W.); Haohaoyue@mail.dlut.edu.cn (H.H.); hufangyuan@dlut.edu.cn (F.H.); liuch1115@dlut.edu.cn (C.L.); chenyousi@dlut.edu.cn (Y.C.); 2 School of Materials Science and Engineering, Dalian University of Technology, Dalian 116024, China; 3 Liaoning Province Engineering Centre of High-Performance Resins, Dalian University of Technology, Dalian 116024, China; 4 State Key Laboratory for Modification of Chemical Fibers and Polymer Materials, Donghua University, Shanghai 201620, China

**Keywords:** carbon fiber, thermoplastic composites, interface/interphase, graphene, silane

## Abstract

This study focused on the faint interface bonding between carbon fiber (CF) and poly(phthalazinone ether ketone) (PPEK) thermoplastic, a multistage hybrid interface layer was constructed via the condensation reaction of N-[3-(Trimethoxysilyl)propyl]-N,N,N-trimethylammonium chloride (KHN^+^) and the electrostatic adsorption of graphene oxide (GO). The influence of the contents of GO (0.2 wt%, 0.4 wt%, 0.6 wt%) on the interfacial properties of composites was explored. FTIR, Raman spectra, XPS tests indicated the successful preparation of CF-KHN^+^-GO reinforcements. The multistage hybrid interface layer significantly increased fiber surface roughness without surface microstructure destruction. Simultaneously, polarity and wettability are remarkably improved as evidenced by the dynamic contact angle experiment. The interlaminar shear strength (ILSS) and flexural strength of the CF/PPEK composites with 0.4 wt% GO (CF-KHN^+^-4GO) were 74.57 and 1508 MPa, which was 25.2% and 23.5% higher than that of untreated CF/PPEK composite, respectively. Dynamic mechanical analysis proved that CF/GO/PPEK composites have excellent high-temperature mechanical properties. This study furnishes an unsophisticated and valid strategy to build an interface transition layer with a strong binding force, which would offer a new train of thought in preparing high-performing structural composites.

## 1. Introduction

Continuous carbon fiber (CF) reinforced polymer composites has been far and wide used as high-performance structural materials in the aerospace industry, nuclear industries, automobiles, machinery, and chemical industries for possessing a combination of commendable properties, such as low density, high strength, strong impact toughness, fatigue endurance [1,2,3,4,5]. In particular, continuous carbon fiber reinforced thermoplastic (CFRTP) composites have many unique advantages, such as high toughness, fatigue resistance, rapid production, melting welding, green recycling, long-term storage of raw materials. In recent years, CFRTP has been applied to large components such as aircraft skin, fairing, wings, and vertical tail, and developed rapidly [6].

It is well-known that the interface of the composite is the bridge between fibers and matrix, which affects the load transfer and crack initiation and propagation [7]. Excellent interface properties can be reduced stress concentration and improve mechanical properties and thermal resistance for the composites [8]. Two main reasons lead to the poor interface bonding of CFRTP. On the one hand, the skin layer in the two-dimensional “skin-core” structure in carbon fiber composed of regular graphite structure possesses a smooth surface and low chemical activity. On the other hand, thermoplastic is highly chemically inert, and no chemical reaction occurs during the hardening process. Therefore, considerable studies have been concentrating on the surface treatments of CF to heighten the interfacial bonding of CFRTPs [9,10,11]. Plasma treatment [12], high-energy irradiation [13], sizing coatings [14,15], chemical grafting [16] techniques, etc., bring forth ways and means to settle the weak interface adhesion of CFRTP sectors.

Graphene oxide (GO), a single layer of graphite oxide bearing oxygen functional groups on its basal side and boundary zone, has already attracted tremendous interest in the domain of polymer composite science on account of its plentiful oxygen-containing functional groups [17,18]. Compared to other carbon fillers, such as carbon nanofibers and carbon nanotubes, its high special surface area, excellent wettability, low cost, large-scale preparation, and remarkable mechanical properties make GO the optimal material for next-generation hierarchical reinforcements of polymer composites [10,19,20]. Recently, some studies have been devoted to chemically grafting GO onto the CF surface to realize the interface enhancement. Huang et al. [21] reported the controllable particle size graphene oxide (GO) sheets were grafted on the CF applying poly(oxypropylene) diamines (D400) as the bridging agent, which led to interlaminar shear strength improving by 34%. Liu et al. [22] grafted GO onto CF by covalent bond composited with PEEK, which led to flexural strength increasing from 762 MPa to 981 MPa. However, it is difficult to evenly graft GO onto CF surface because of the reactivity and steric hindrance, which is easy to form stress concentration at the interface phase [23]. Physical surface coating could achieve the goal of uniform distribution of graphene carbon fiber surface. GO attach to CF based on physical interactions and Van der Waals forces, which are propitious to nanoplatelets peeling from CF surface, followed by interface delamination. To sum up, the uniform distribution of GO nanosheets on the CF surface through strong binding force is the key to maximize the interface enhancement efficiency of GO. The physical surface coating has no negative effects on the structure and performance of carbon fiber. Nevertheless, aside from sizing, physical surface coating is hardly investigated in the literature. The thermosetting sizing agents are the most commonly used sizing agents, however, with poor compatibility with thermoplastic matrix [24,25,26]. This incompatibility leads to poor mechanical strength of CFRTP, especially in terms of interlaminar shear strength (ILSS). Sizing agent doped nanoparticles can improves the compatibility between sizing agents and thermoplastic matrix, but also cause agglomeration [14,27,28].

Poly(phthalazinone ether ketone) (PPEK), as a promising thermoplastic matrix, possesses excellent heat-resistant, prominent mechanical property and unique solubility owing to its twisted and noncoplanar macromolecular chain structures. Its glass transition temperature (*T_g_*) can reach up to 263 °C, which is much higher than poly (ether ketone) (PEEK, *T_g_* = 143 °C), poly(phenylene sulfide) (PPS, *T_g_* = 85 °C) and polysulfone (PSU, *T_g_* = 185 °C) [16,29] and other traditional high-performance thermoplastic. In addition, PPEK can be dissolved in organic solvents such as chloroform, 1-Methyl-2-pyrrolidinone (NMP), dimethylacetamide (DMAc) under room temperature, which overcomes the insoluble shortcomings of traditional thermoplastic resins and realizes the preparation of prepregs by solution impregnation. Nevertheless, PEEK and PPS can only dissolve in concentrated sulfuric acid, and their composites can only be fabricated by melt impregnation. The method usually involves high cost and energy consumption [24].

Given the above, in order to improve the interface performance of CF/PPEK composite, GO/CF multi-scale reinforcement was prepared by electrostatic interaction. First, the abundant hydroxyl groups on the carbon fiber surface reacted with the positively charged silane coupling agent, N-trimethoxysilylpropyl-N,N,N-trimethylammonium chloride (KHN^+^), to form a positively charged silane coating on CF surface. Noteworthily, silane layer with plentiful hydroxyl groups is easily prepared via the gradual hydrolysis and condensation of multifunctional silicon sources such as tetraethoxysilane and silane coupling agents. In our previous study, the silane layer contained POSS is beneficial to the interface performance and hygrothermal aging resistance of CFRTP [2,30]. Second, GO bearing a large number of carboxyl groups can be ionized in water. The positively charged CF coated by silane is put into an aqueous dispersion of GO, and then the GO nanosheets could be adsorbed on CF surface by electrostatic action. The electrostatic force between CF and GO is far stronger than Van der Waals forces. Furthermore, the GO nanosheets can be evenly distributed onto the CF surface because of the equal distribution of positive charge in the silane coating. The force of electrostatic adsorption is much greater than other physical adsorption methods [31] and the electrostatic adsorption method is a non-destructive method that there is no need to process the fiber by ionization, acidification, etc. [32,33]. The investigation showed a very remarkable improvement of interfacial interlocking proved by the mechanical properties and the morphology of fractured surfaces, and the interlaminar shear strength.

## 2. Experiment

### 2.1. Materials

**Figure 1:**
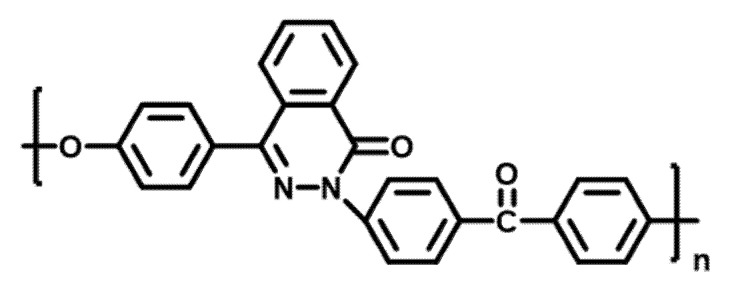
Molecular structure of PPEK.

PPEK resin, the molecular structure is shown in Figure 1, were purchased from Dalian Polymer New Material Co., Ltd., Liaoning, China with intrinsic viscosity [η] of 0.80 dL/g. The commercially available continuous carbon fiber used in this study was T700 (12 K) with an average diameter of 7 µm, obtained from Toray Industries, Inc. Graphene Oxide gel and N-[3-(Trimethoxysilyl)propyl]-N,N,N-trimethylammonium chloride were purchased from Aladdin Bio-Chem Technology Co., Ltd. (Shanghai, China). All solvents and other reagents were provided by commercial sources and used as received.

### 2.2. Preparation of KHN^+^ Silane Functionalized CF

**Figure 2:**
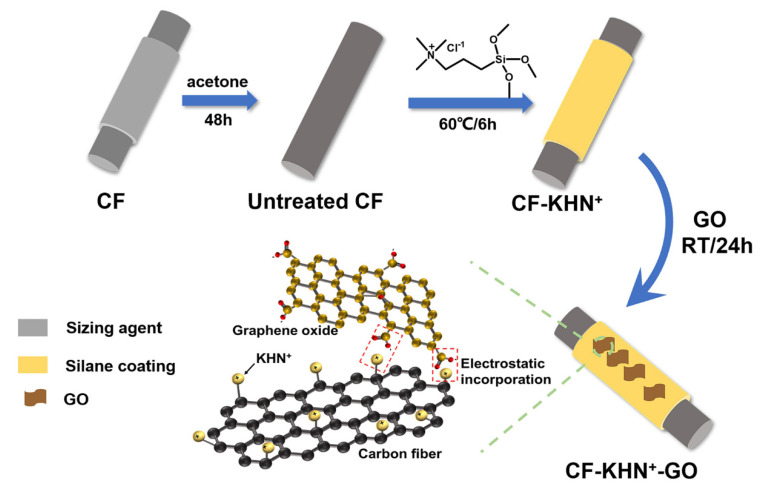
The schematic diagram of fabrication GO/CF multi-scale reinforcement.

The CF bundles were refluxed with ethyl alcohol and acetone for 48 h and then dried in a vacuum oven at 80 °C to eliminate the interference factor of commercial size and remove dust, grease, named Untreated CF. Then, 4 mL of N-[3-(Trimethoxysilyl)propyl]-N,N,N-trimethylammonium chloride (KHN^+^) were dissolved into 50 mL methanol. Then, 5 g CF wrapped around a glass frame were added into the above solution and stirred at 60 °C for 6 h. Subsequently, the modified CF were washed repeatedly with ethanol, dried in an oven at 80 °C for 4 h, which was denoted as CF-KHN^+^ (Figure 2).

### 2.3. Preparation GO/CF Muti-Scale Reinforcements

A quantitative amount of GO was added to deionized water, sonicated for 1 h (Granbo Technology Industrial Shenzhen Co., Ltd., 450 W, 40 kHz), and configured into a graphene oxide aqueous suspension with content of 0.2 wt%, 0.4 wt%, and 0.6 wt%, respectively. Then, the CF was immersed in the suspension, and stand for 24 h. Finally, the obtained CF were dried in an oven at 80 °C for 4 h, and denoted as CF-KHN^+^-2GO, CF-KHN^+^-4GO, and CF-KHN^+^-6GO.

### 2.4. Preparation of CF/PPEK Composites

CF/PPEK composites were prepared through the parallel solution impregnation process [2]. First, the CF was wound on a 250 mm × 150 mm iron frame, and 13 g PPEK resin were dissolved in 100 mL of NMP (13 wt%). Then, the CF-wrapped iron frame was immersed in the PPEK/NMP resin solution. After being fully soaked, the iron frame wrapped with CF was placed in a blast oven for drying. The drying system was 100 °C/2 h, 120 °C/2 h, 150 °C/2 h, 180 °C/2 h, 280 °C/2 h. Finally, the CF reinforced PPEK resin unidirectional tape was cut into 100 mm × 60 mm prepregs. Fourteen piles of neatly tailored prepreg plies were stacked unidirectionally into a steel mold with the pressure of 5 MPa at 350 °C for 2 h.

### 2.5. Characterization Methods

Thermogravimetric analyses (TGA) of the polymers were performed on a Mettler TGA/SDTA851 thermogravimetric analysis instrument heating from 30 °C to 800 °C under nitrogen atmosphere at a heating rate of 20 °C/min. The differential scanning calorimetric (DSC) was measured on Mettler DSC822 under a nitrogen flow (50 mL/min) heating rate of 20 °C/min from 30 to 350 °C. The TGA and DSC curves of PPEK and CF/PPEK composites were presented in Figure S3.

FTIR spectrophotometer was performed with ThermoFisher, 6700 (USA), which was applied to characterize the chemical structure of different CF. The specimens were prepared with powder-pressed KBr pellets. FTIR tests were carried out scanning the specimens 64 times in the wavenumber range of 400–4000 cm^−1^ with the resolution of 16 cm^−1^.

XPS was used to detect surface composition and analyze the functionalization reaction mechanisms. The spectra were collected using a Thermo Fisher Scientific (ESCALAB250Xi, UK) with a monochromatic Al Ka source (1486.6 eV) at base pressure of 2 × 10^−9^ mbar.

Raman spectroscopy from 500 to 4000 cm^−1^ was used an Ar^+^ laser (wavelength is 514.5 nm) at room temperature by Invia Renishaw 2000 spectrometer. Raman focusing and imaging was conducted using a confocal microscope with an objective of 50× and the spot diameter of 1–2 µm.

The surface morphology of CF according to different modifications and general analyses for the material after mechanical tests of CF/GO/PPEK composites were observed by SEM (Japan Hitachi Nake high-tech enterprise, SU8220). All the samples were coated with a thin conductive gold layer before SEM measurements. The surface roughness of different reinforcements was determined by a dimension icon atomic force microscope (AFM) (Bruker, Dimension Icon)

A dynamic contact angle metre and tensiometer (DCAT21, Data Physics Instruments, Germany) were used to characterize the dynamic contact angle and surface energy. Deionized water (*γ^d^* = 21.8 mN/m, *γ* = 72.8 mN/m^−1^) and diiodomethane (*γ^d^* = 50.8 mN/m, *γ* = 50.8 mN/m, 99% purity, Alfa Aesar, Ward Hill, NY, USA) were applied as the test liquids. The dispersive and polar components of the hybrid CF were calculated according to Equations (1) and (2) [34,35]:

(1) $$\gamma_{1}\left(1=\cos\theta \right)=2{\left(\gamma_{1}^{p}\gamma_{f}^{p}\right)}^{\frac{1}{2}}+2{\left(\gamma_{1}^{d}\gamma_{f}^{d}\right)}^{\frac{1}{2}}$$

(2) $$\gamma_{f}=\gamma_{f}^{p}+\gamma_{f}^{d}$$

where $\gamma_{1}$, $\gamma_{1}^{d}$ and $\gamma_{1}^{p}$ are the surface tension of the test liquid, its dispersive and polar component, respectively.

The interlaminar shear strength (ILSS) (see Figure S1 for schematic diagram) of the CF/PPEK composites were performed on Instron 5982 Universal test machine (Boston, MA, USA). The specimen dimensions were 20 mm × 10 mm × 2 mm and the specimens were measured at the cross-head speed of 1 mm/min, according to the ISO 14130 standard. At least 5 samples were tested for each processing condition. The *ILSS* values were calculated according to Equation (3):

(3) $$ILSS=\frac{3P_{b}}{4bh}$$

where *P_b_* is the maximum compression load at fracture (N), *b* is the width of the specimen (mm), and *h* is the thickness of the specimen (mm).

The flexural strength of the CF/PPEK composites (see Figure S2 for schematic diagram) was tested according to ASTM D790-10 using a three-point flexural test method. The size of the specimen was 80 mm × 12.5 mm × 2 mm, and they were tested at the crosshead movement rate of 2 mm/min, with the span of 64 mm. At least five measurements were performed for each composite and get the average values of flexural strength and modulus.

Dynamic mechanical analysis (DMA) tests can obtain the dynamic mechanical spectra (tan δ, storage modulus, loss modulus). DMA were carried out with a TA Q800 instrument at 1 Hz and the heating rate of 3 °C/min under a single cantilever mode to characterize *T_g_*. The test temperature ranged from 25 °C to 300 °C. The composite sample dimensions were 35 mm × 6 mm × 2 mm for the composite samples. In DMA, a complex modulus (G*), an elastic modulus (G′), and an imaginary (loss) modulus(G″) are calculated from the material response to the sine wave. These different moduli allow better characterization of the material, because we can now examine the ability of the material to return or store energy (G′), to its ability to lose energy (G″), and the ratio of these effects (tan delta), which is called damping.

(4) $$G^{*}=G^{\prime }+iG^{\prime \prime }$$

(5) $$\tan\delta =G^{\prime \prime }/G^{\prime }$$

## 3. Results and Discussion

### 3.1. Surface Structures and Chemical Elements of CF

**Figure 3:**
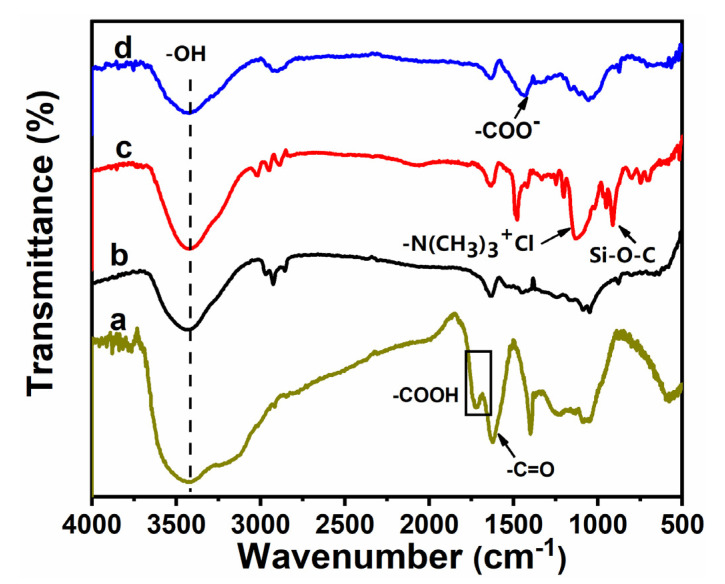
FTIR spectra of carbon fiber: (**a**) GO, (**b**) Untreated CF, (**c**) CF-KHN^+^, (**d**) CF-KHN^+^-4GO.

The FTIR spectra of GO, untreated CF, CF-KHN^+^, and CF-KHN^+^-4GO were shown in Figure 3. The absorption peak of GO chiefly consists of stretching vibrations of O-H from the hydroxyl and carboxyl groups at 3400 cm^−1^. The stretching bands at 1729 cm^−1^ locate C=O from carboxyl. This indicates that a large number of oxygen-containing functional groups exist on the GO surface. The absorption peak in the range of 3250~3450 cm^−1^ corresponds to the -OH stretching vibration peak of the CF surface. The characteristic bands at 952 cm^−1^ and 1141 cm^−1^ in CF-KHN^+^ spectrum are ascribed to the stretching vibration of Si-O-Si and Si-OH, indicating the existence of silane coating on the CF-KHN^+^ surface. After introducing GO, the symmetric stretching of the carboxylate group appeared at 1420 cm^−1^, which can be attributable to the abundant carboxyl groups on the graphene oxide. It shows that graphene oxide has been introduced to the surface of the fiber.

**Figure 4:**
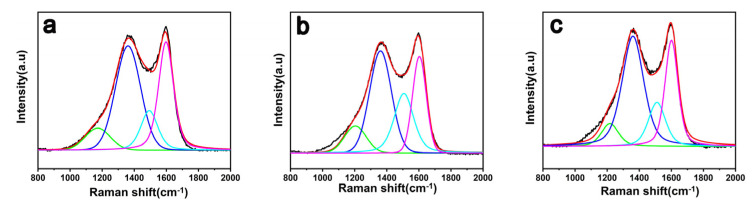
Raman spectra of carbon fiber (**a**) Untreated CF, (**b**) CF-KHN^+^, (**c**) CF-KHN^+^-4GO.

Figure 4 shows Raman spectra of three kinds of CF surface. The Raman spectrum of CF has two distinct characteristic peaks, which are the D-band absorption peak at about 1360 cm^−1^ and the G-band absorption peak at 1600 cm^−1^. Peak D represents the defect amorphous carbon structure on the surface of the CF, corresponding to sp^3^ hybridized carbon atoms; Peak G represents the regular and ordered graphitized carbon structure on the surface of the CF, corresponding to sp^2^ hybridized carbon atoms [36]. The integrated intensity ratio (AD/AG) is an important indication of the degree of graphitization of the CF surface. The larger value of AD/AG, the higher the degree of disorder on the CF surface [37]. Perform peak fitting of Raman spectrum, and new peaks T and A appear at 1160 and 1510 cm^−1^, respectively [38]. The values of AD/AG are shown in Table 1.

The Raman parameters of the modified CF have undergone certain changes. The Raman peaks of the D peak and the G peak are slightly blue-shifted, and the integrated intensity ratio AD/AG rises from 2.29 (Untreated CF) to 2.70 (CF-KNH^+^). It indicated that the number of sp^3^ hybrid carbon atoms on the CF-KNH^+^ surface increases with the modification reaction and the disorder of the CF-KNH^+^ surface has the same trade. Meanwhile, the result further proves that the CF surface have been introduced with a silane coating. With the GO contained negative charge adding, the AG/AD increases to 3.38, the degree of disorder on the CF-KNH^+^-4GO surface is further improved, and the reaction activity is further increased.

**Figure 5:**
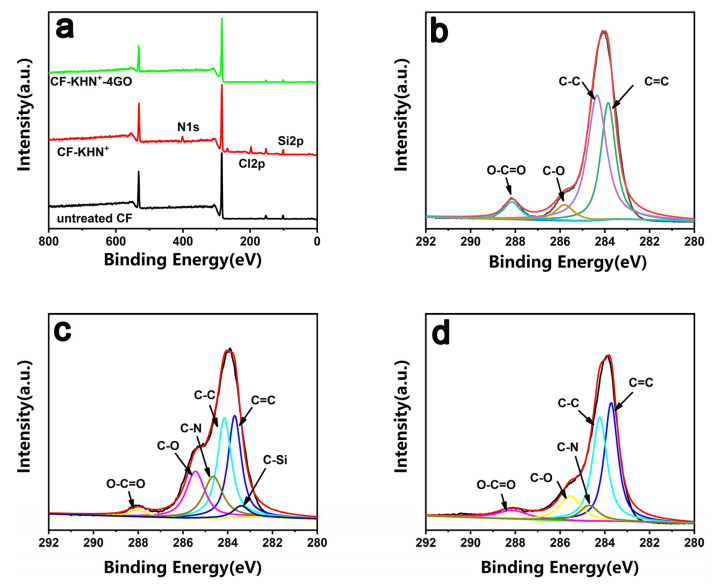
XPS full spectra of carbon fibers (**a**) and XPS curve fitting of carbon fibers. (**b**) Untreated CF, (**c**) CF-KHN^+^, (**d**) CF-KHN^+^-4GO.

XPS was used to analyze the changes of surface element content and chemical composition before and after CF modification. Figure 5a shows the XPS wide-scan spectra of various CF. Untreated CF is mainly composed of carbon and oxygen. After constructing a silane coating contained negative charge on the CF-KNH^+^ surface, the characteristic peaks of Si2p (109 eV) and N1s (399 eV) elements initially appear on the CF-KNH^+^ surface. It proves that the silane coating contained negative charge is successfully constructed on the CF-KNH^+^ surface. After adsorbed GO, the content of N element and Si element drops sharply. To better reveal the modification process, high resolution spectra of C1s were analyzed. Compared with the untreated CF, CF-KHN^+^ has two new peaks at 284.6 eV and 284.3 eV ascribed to the C-N and C-Si, as shown in Figure 5c. This result proves that the construction of silane coating contained negative charge is successfully constructed on the CF-KHN^+^ surface. For CF-KHN^+^-4GO, it can be observed in the spectrum that the C-N bond decreases and the C-Si bond disappears. Additionally, the enhancements of O-C=O contents could be attributed to the introduction of GO nanosheets.

### 3.2. Surface Morphology of CF

**Figure 6:**
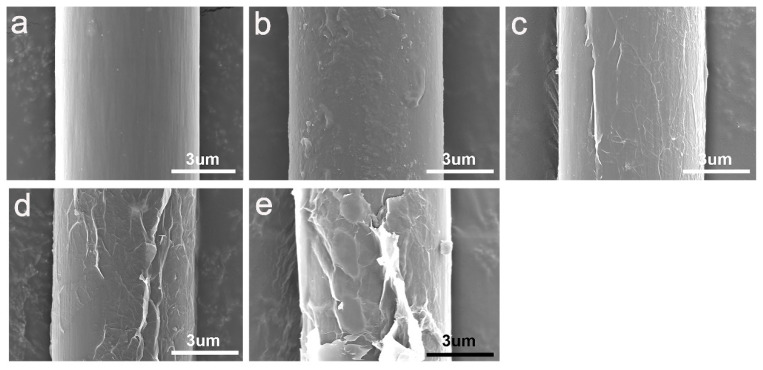
SEM images of carbon fibers (**a**) Untreated CF, (**b**) CF-KHN^+^, (**c**) CF-KHN^+^-2GO, (**d**) CF-KHN^+^-4GO, (**e**) CF-KHN^+^-6GO.

As shown in Figure 6, SEM was used to observe the surface morphology and surface characteristics of different CF. The surface of untreated CF is relatively smooth and clean (Figure 6a). Some shallow grooves left during the preparation process can be observed. These grooves were parallel arranged along with the axial direction of CF. Compared with untreated CF, the silane coating on the surface of CF-KNH^+^ filled the grooves on the fiber surface and formed new wrinkles.

Remarkably, differences of the surface morphology can be observed after absorbing GO, as shown in Figure 6c–e. In order to further study the influence of graphene oxide content on surface morphology, three multi-scale reinforcements (CF-KHN^+^-2GO, CF-KHN^+^-4GO, CF-KHN^+^-6GO) were prepared. GO is attached onto the CF-KHN^+^ surface, however, the density of GO is different. It is easy to understand that CF have a high GO density after being treated with high concentration of GO aqueous suspension. In Figure 6c, only a small amount of GO could be observed attached onto the surface of CF-KHN^+^-2GO. The foremost reason is that the content of GO is little, which limits its adsorption onto the positively charged silane coating. As shown in Figure 6d, the entire CF-KHN^+^-4GO surface was homogeneously covered with GO nanosheets. However, a large number of GO nanosheets were concentrated onto the CF-KHN^+^-6GO surface (Figure 6e), and forming huge wrinkles and big blocks. Superfluous GO nanosheets lead to aggregation/agglomeration phenomena that can generate stress concentrations and decrease the energy dissipation capability, which result in less effective interfacial enhancement of its composite.

**Figure 7:**
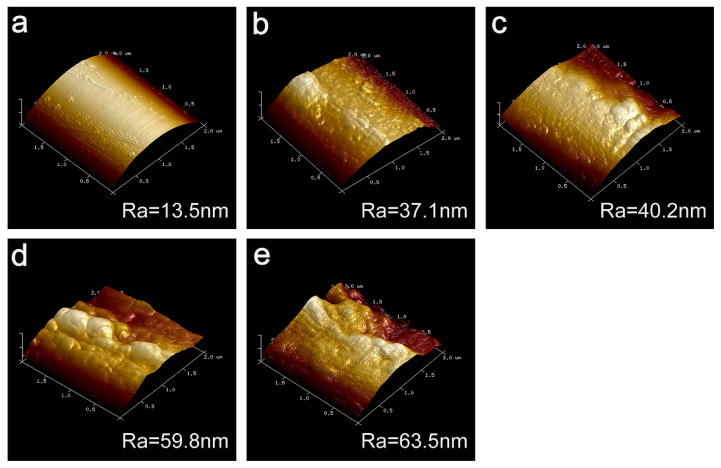
AFM images of carbon fibers (**a**) Untreated CF, (**b**) CF-KHN^+^, (**c**) CF-KHN^+^-2GO, (**d**) CF-KHN^+^-4GO, (**e**) CF-KHN^+^-6GO.

The surface roughness (Ra) was also quantitatively characterized by AFM. Compared with the untreated CF, the CF-KHN^+^ surface roughness has also been increased from 13.5 nm (Figure 7a) to 37.1 nm (Figure 7b). Its noteworthiness is that the surface roughness of CF-KHN^+^-2GO (Ra = 40.2 nm) has no significant change. The main reason is that the GO content is too low, and the adsorption amount is small. The fiber surface roughness of CF-KHN^+^-4GO increased from 40.2 nm to 59.8 nm with a large margin and the GO nanosheets were distributed uniformly in different directions. Moreover, The GO played a role of locking for enhancing the interfacial adhesion between the fiber and the matrix by inserting into the composite interface region. CF-KHN^+^-6GO has a larger roughness of 63.5 nm. Nevertheless, it could be found from (Figure 6e) that a large amount of GO aggregated onto the fiber surface, which is not conducive to enhance interfacial strength between the fiber and the matrix caused by stress concentration.

### 3.3. Surface Free Energy Analysis and Wettability of CFs

Contact angle (*θ*) and surface energy (*γ*) of different reinforcements including dispersion component (*γ^d^*) and polar component (*γ^p^*) are summarized in Table 2. As known, chemical structure and surface morphology affect the fiber surface energy. The increase of the fiber surface energy can ameliorate the surface wettability and interfacial adhesion between CF and matrix [39]. The surface energy of untreated CF and CF-KNH^+^ is 41.16 mN/m and 45.05 mN/m, respectively. The polar component of untreated CF was lower due to the less polar functional groups, the formation of the silane coating improves the polar groups on the CF surface, and increases the surface energy. After GO was introduced onto the CF surface by electrostatic action, the surface energy presented more distinct increasing tendency, from 41.16 mN/m to 49.46 mN/m. Moreover, with the increase of GO contents, the surface energy increased evidently to 59.09 mN/m for CF-KNH^+^-4GO. The introduction of GO increases the number of polar groups on the fiber surface, and at the same time increases the surface roughness of the fiber. This change of surface energy is mainly caused by the increased polar component (*γ^p^*). The higher surface energy could lead to better wettability particularly, which was beneficial for the improvement of the composite’s interfacial properties. The surface energy of CF-KNH^+^-6GO is 57.22 mN/m, which is slightly lower than that of CF-KNH^+^-4GO. The *γ^p^* of the two reinforcements are almost equal, indicating that the content of polar groups on the surface have little difference. The *γ^d^* of CF-KNH^+^-4GO is larger than CF-KNH^+^-6GO because CF-KNH^+^-4GO has preferable interface morphology. Hence, CF-KNH^+^-4GO with the highest surface energy enhances the wettability effectively and improves the interfacial strength.

### 3.4. Interfacial Property Testing of Composites

**Figure 8:**
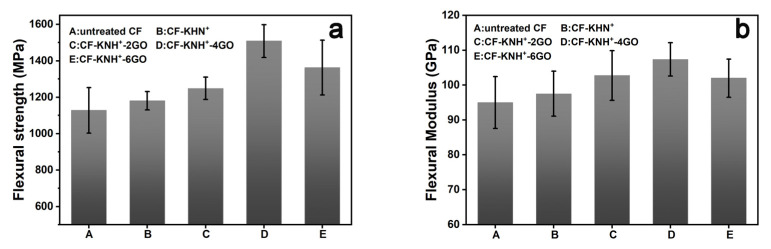
(**a**) Flexural strength of GO/CF multiscale composites and (**b**) Flexural Modulus of GO/CF multiscale composites.

The study of flexural properties adopting three-point bending mode could comprehensively reflect the mechanical performance of CF/PPEK composites involved tensile, compressive and shear forces form frequently met in the engineering applications. As shown in Figure 8, the flexural strength and modulus of untreated CF/PPEK composite are 1128 MPa and 95.00 GPa, whereas flexural strength and modulus of CF-KNH^+^/PPEK composite are 1180 MPa and 97.5 GPa. After KHN^+^ coating, the polar groups onto the CF surface can increase the fiber surface energy, and the wettability with PPEK have been enhanced, which is accountable for the improvement in interfacial bonding properties. The flexural strengths of CF-KNH^+^-2GO/PPEK, CF-KNH^+^-4GO/PPEK, and CF-KNH^+^-6GO/PPEK composites are 1248 MPa, 1508 MPa and 1362 MPa, respectively. The high flexural strength after the surface treatment is mainly owing to the increase of its interface strength. In the interface layer, the electrostatic interaction between the GO nanosheets layer and the CF improves the stress transfer efficiency from the matrix to the reinforcement under the action of environmental stress relieves the stress concentration caused by interface defects and increases the stress bearing capacity. The Stress–Strain curves of untreated CF/PPEK composites and CF-KNH^+^-4GO/PPEK composites were presented in Figure S4. It is still noteworthy that the variation tendency of flexural modulus was the same as that of flexural strength. The flexural modulus of CF-KNH^+^-2GO/PPEK, CF-KNH^+^-4GO/PPEK, and CF-KNH^+^-6GO/PPEK composites were 102 GPa, 107 GPa and 102 GPa, respectively. CF-KNH^+^-4GO/PPEK exhibits the highest strengthening effect (107 GPa). The uniform distribution of GO nanosheets in the interface layer, the best mechanical interlocking and wettability between CF-KNH^+^-4GO and PPEK result in the strongest interface adhesion for CF-KNH^+^-4GO/PPEK composite, which restricts the movement of PPEK molecules at the interface, and leads to the greatest flexural strength and modulus.

**Figure 9:**
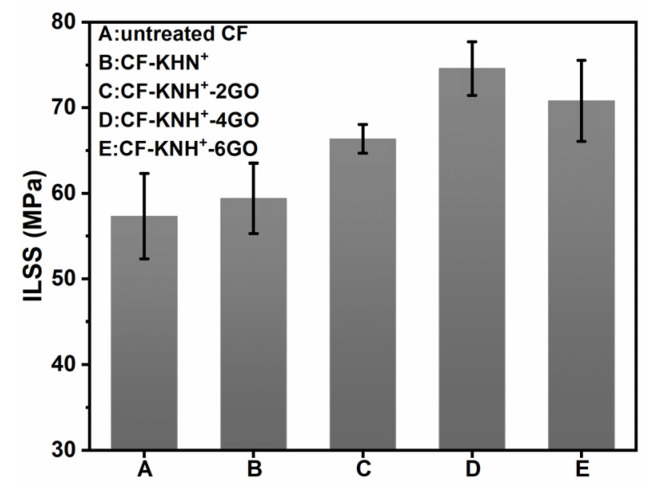
ILSS of GO/CF multiscale composites.

The short beam three-point bending method was utilized to characterize the interlaminar shear strength (ILSS) of the CF/PPEK composites, as shown in Figure 9. The ILSS value of the untreated CF/PPEK composite is 57.00 MPa. As the KNH^+^ were coated onto CF surfaces, CF-KHN^+^/PPEK composite showed the higher interlaminar shear strength (59.69 MPa). The introduction of KNH^+^ onto CF surfaces is beneficial to the infiltration of CF by the resin matrix because it can favor increasing fiber surface energy. Compared with CF-KHN^+^/PPEK composite, the ILSS of CF-KHN^+^-2GO/PPEK composite is 65.33 MPa with 9.45% increment. The introduced 0.2 wt% GO on fiber surface do not ameliorate ILSS efficaciously which is may due to the poor wettability as well as weak mechanical interlock between GO and PPEK. When the concentration of graphene oxide is 0.4 wt%, GO is distributed on fiber surface without significant agglomeration. The interface area effectively hardening favors improve the stress transfer efficiencies. Hence, ILSS of CF-KHN^+^-4GO/PPEK composite remarkably increases to 74.57 MPa. Relative decrease of the ILSS value of CF-KHN^+^-6GO (70.79 MPa) was observed due to the agglomeration of GO in the interface region and that has been confirmed by Figure 6e. The change rule of mechanical properties is the same as SEM and AFM image analysis.

### 3.5. Fractured Surface Morphology of Composites

**Figure 10:**
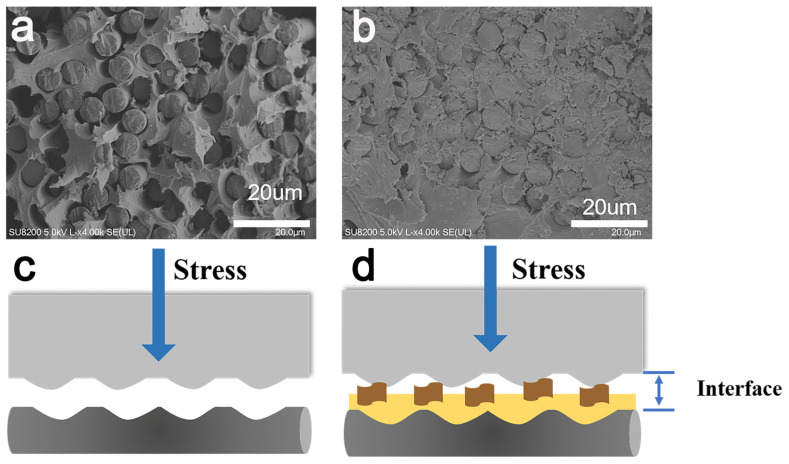
SEM morphologies of the flexural fracture surface of (**a**) Untreated CF/PPEK; (**b**) CF-KHN^+^-4GO/PPEK; Proposed failure mechanism of composites: (**c**) Untreated CF/PPEK, (**d**) CF-KHN^+^-4GO /PPEK.

To determine the interface enhancement mechanism for CF-KHN^+^-4GO/PPEK composite, the cross-sectional morphologies of the untreated CF composite and CF-KHN^+^-4GO composite were verified by SEM, as shown in Figure 10, for untreated CF composite (Figure 10a), a lot of CF were pulled out from the PPEK, and some holes remained in the PPEK matrix as a result of the weak interface combination between the untreated CF and PPEK. The interfacial property of CF-KHN^+^-4GO/PPEK composite improved significantly. The fiber debonding, holes, and pulled-out fibers on the CF-KHN^+^-4GO/PPEK composite fractured surface have not been found.

The microscopic fracture mechanism was seen to be interfacial adhesion failure and the schematic diagrams were displayed in Figure 10c,d. The untreated CF has low surface roughness (Ra = 13.5 nm) and low surface energy (41.16 mN/m) (Figure 7a and Table 2). PPEK with high viscosity is difficult to infiltrate completely untreated CF bundles, which would cause gaps, holes and other defects between the fibers and the PPEK matrix. Under stress, cracks propagate along the interface and gradually develop leading to interface failure. When the carbon fiber was modified by GO nanosheets, the mechanical interlock between CF-KHN^+^-4GO and PPEK matrix is strengthened by the increase of the fiber surface roughness. Owing to the existence of GO nanosheets, the crack is blunted and turned to prevent the crack from propagating. With the increase of external force, the PPEK resin in the composite material yields and even the carbon fiber breaks.

**Figure 11:**
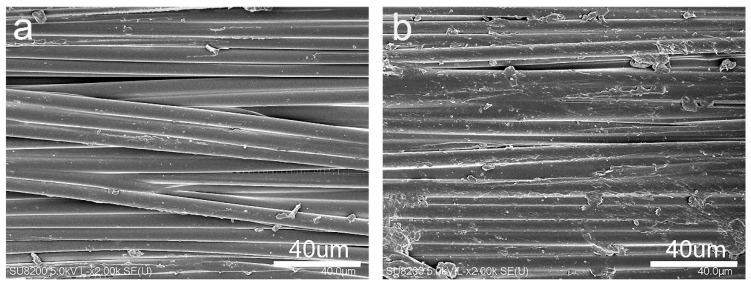
SEM morphologies of ILSS fracture surface of composite: (**a**) Untreated CF and (**b**) CF-KHN^+^-4GO.

Figure 11 shows the SEM morphologies of the ILSS fracture of different composite samples. For untreated CF composite (Figure 11a), a large number of bare CF are exposed, a typical feature of interface failure. Many voids and cracks are observed in untreated CF/PPEK. Fibers with a smooth surface were debonded from the PPEK matrix, suggested that the adhesive failure was initiated along fiber surface due to the poor interfacial bonding. However, for the CF-KHN^+^-4GO/PPEK composite (Figure 11b), PPEK matrix layer covers on fibers, and some fragmented resins were adsorbed on the fiber surface. The CF-KHN^+^-4GO/PPEK composite showed typical matrix failure characteristics under shear force [40,41], the fracture occurred at the matrix because the interfacial strength between the fiber and the matrix was improved by absorbing GO. The fracture model was changed from pure fibers broke to the combination failures of fibers broke, interface and delamination [42,43]. Therefore, GO nanosheets significantly improve the interface bonding between the fibers and the matrix by increasing surface roughness and polarity of CF.

**Figure 12:**
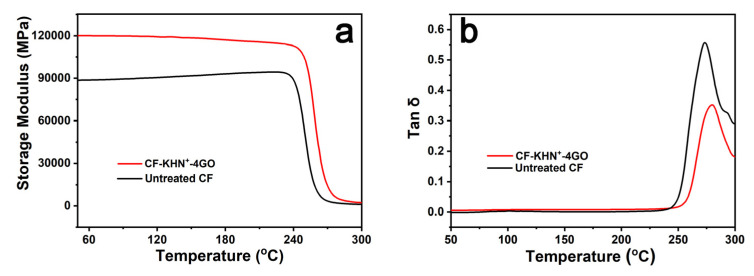
Temperature dependence of untreated CF/PPEK and CF/GO/PPEK composites: (**a**) storage modulus, and (**b**) loss tangent delta.

Dynamic mechanical analysis (DMA) has been generally applied to characterize the interfacial adhesion of assorted polymeric systems. The temperature dependence of the storage modulus for different composites are shown in Figure 12a. The initial modulus of CF-KHN^+^-4GO/PPEK composite is about 120 GPa at room temperature, which is surmounted that of untreated CF/PPEK composite (90 GPa). The silane/GO hybrid rigid interface layer increases the thickness of the interface phase and the volume content of the reinforcement in the composite material. This result is in good agreement with that of flexural modulus.

It is well-known that *T_g_* can be defined as the max value of tanδ with a low frequency. Figure 12b shows that the untreated CF/PPEK composite has a *T_g_* of 277 °C, and the *T_g_* of CF-KNH^+^-4GO/PPEK composite is approximately 9 °C higher than its counterpart. The silane/GO hybrid rigid interface layer restricts the movement of the matrix resin near the interfacial phase. Additionally, the loss factor tanδ reflects the balance of elasticity and viscosity in the composite. With the loss factor tanδ increasing, the viscosity of the composite increases and the elasticity decreases. The weaker the interface bonding of the composite, the larger the loss factor tanδ [30]. The interface bonding of untreated CF/PPEK composites is weak, and the loss factor tanδ peak value is 0.57. After silane/GO modification, the interface bonding between CF-KNH^+^-4GO and PPEK enhanced slightly, the loss factor tanδ peak value of CF-KNH^+^-4GO/PPEK composite dropped to 0.36, indicating that it has good interfacial property.

## 4. Conclusions

In this study, the silane/GO hybrid interface layer were constructed to enhance the interface adhesion of CF/PPEK. The results of FTIR, Raman, and XPS affirmed the successful preparation of CF-KNH^+^-4GO reinforcement. The SEM and AFM results manifest that the roughness increased obviously through electrostatic adsorbing GO. The surface energy of CF is also significantly increased with the addition of GO. The optimal GO content was 0.4 wt%, with ILSS of 74.57 MPa and flexural strength of 1508 MPa. Based on the mechanical properties investigation, the silane/GO hybrid interface layer improves the interfacial bonding strength of composite. The content of graphene oxide affects the bonding strength between fiber and resin. CF-KHN^+^-4GO exhibits the best interface enhancement effect, with ILSS of 74.57 MPa and flexural strength of 1508 MPa. The reinforcing mechanisms were also elaborated that the GO sheets play the anchoring effects in interfacial area. Moreover, this method can be conveniently operated and would have great potential for using as multi-scale reinforcements in high-performance composites.

## Figures and Tables

**Table 1:** Wavenumbers of D, G and A bands and the calculated values of “AD/AG” acquired from carbon fibers.

	D	G	A	
Samples	W (cm^−1^)	W (cm^−1^)	W (cm^−1^)	R = AD/AG
Untreated CF	1359	1596	1512	2.29
CF-KNH^+^	1363	1601	1525	2.70
CF-KNH^+^-4GO	1364	1602	1529	3.38

**Table 2:** Dynamic contact angles and surface energies of different reinforcements.

	Contact Angle *θ* (°)		*γ^d^* (mN/m)	*γ^p^* (mN/m)	*γ* (mN/m)
	Water	Diiodomethane			
Untreated CF	69.8	56.3	30.70	10.46	41.16
CF-KNH^+^	65.4	52.1	33.10	11.95	45.05
CF-KNH^+^-2GO	61.7	44.9	37.06	12.4	49.46
CF-KNH^+^-4GO	50.2	34.7	42.17	16.92	59.09
CF-KNH^+^-6GO	50.6	35.8	41.66	16.83	57.22

## Data Availability

Data sharing is not applicable for this article.

## Supplementary Materials

The following are available online at https://www.mdpi.com/article/10.3390/ma15010206/s1, Figure S1: Schematic diagram of interlaminar shear experiment, Figure S2: Schematic diagram of flexural experiment, Figure S3: (a) TGA Spectra of PPEK and CF-KHN+-4GO/PPEK; (b) DSC Spectra of PPEK and CF-KHN+-4GO/PPEK.
