# Supplementary material for: Establishment of Silane/GO Multistage Hybrid Interface Layer to Improve Interfacial and Mechanical Properties of Carbon Fiber Reinforced Poly (phthalazinone ether ketone) Thermoplastic Composites
Source: Materials (Basel). 2021 Dec 28;15(1):206. doi: 10.3390/ma15010206 (PMC8745983; doi:10.3390/ma15010206)
Supplement: Supplementary file 1 [file materials-15-00206-s001.zip › materials-1419699-supplementary.pdf]

materials

Supplementary materials.

Establishment of Silane/GO Multistage Hybrid Interface Layer to Improve Interfacial and Mechanical Properties of Carbon Fiber Reinforced Poly (phthalazinone ether ketone) Thermoplastic Composites

Shan Cheng [1,2,4], Nan Li [1,3,4,*], Yuxi Pan [1,4], Bing Wang [1,2,4], Haoyue Hao [1,2,4], Fangyuan Hu [1,2,4], Cheng Liu [1,4], Yousi Chen [1,4] and Xigao Jian [1,2,4,*]

[11] State Key Laboratory of Fine Chemicals, Department of Polymer Science and Materials, School of Chemical Engineering, Dalian University of Technology, Dalian 116024, China; 15610331427m@sina.cn (S.C.); pan.yuxi@163.com (Y.P.); 17864301035@163.com (B.W.); Haohaoyue@mail.dlut.edu.cn (H.H.); hufangyuan@dlut.edu.cn (F.H.); liuch1115@dlut.edu.cn (C.L.); chenyousi@dlut.edu.cn (Y.C.)
[2] School of Materials Science and Engineering, Dalian University of Technology, Dalian 116024, China
[3] State Key Laboratory for Modification of Chemical Fibers and Polymer Materials, Donghua University, Shanghai 201620, China
[4] Liaoning Province Engineering Centre of High-Performance Resins, Dalian University of Technology, Dalian 116024, China
* Correspondence: polymerlinan@dlut.edu.cn (N.L.); jian4616@dlut.edu.cn (X.J.); Tel.: +86-411-84986092

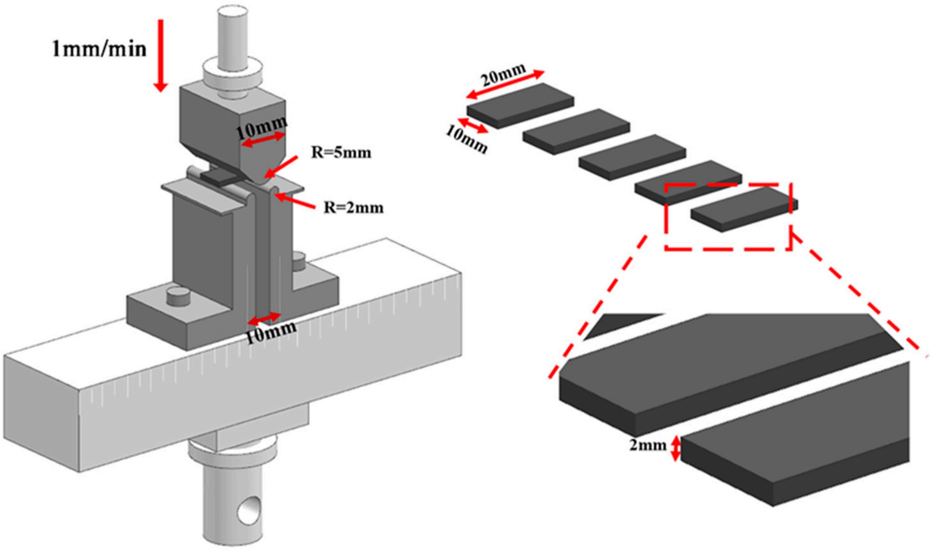

Figure S1. Schematic diagram of interlaminar shear experiment.

The instrument and specimen used for interlaminar shear strength (ILSS) are shown in Fig.S1. The ILSS of the CF/PPEK composites were performed on Instron 5982 Universal test machine (Boston, Massachusetts, USA). The specimen dimensions were 20 mm × 10 mm × 2 mm and the specimens were measured at the cross-head speed of 1 mm/min, according to the ISO 14130 standard. At least 5 samples were tested for each processing condition. The ILSS values were calculated according to Equation:

$$ILSS = \frac{3P_b}{4bh} \tag{1}$$

where P_b is the maximum compression load at fracture (N), b is the width of the specimen (mm), and h is the thickness of the specimen (mm).

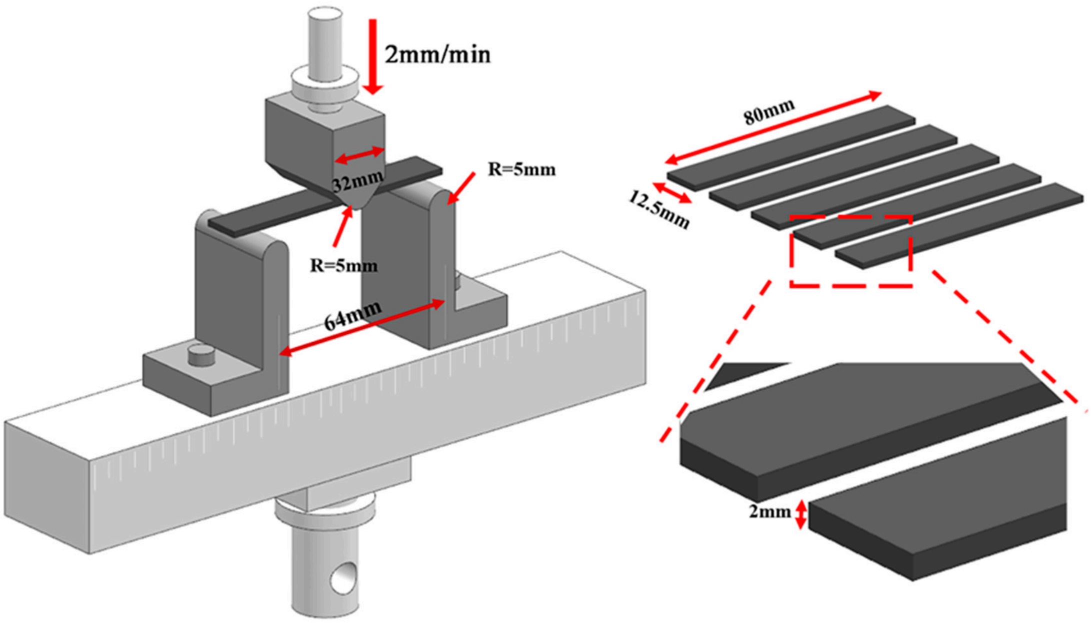

Figure S2. Schematic diagram of flexural experiment.

Fig.S2 shows the schematic diagram of flexural experiment. The flexural strength of the CF/PPEK composites was tested according to ASTM D790-10 using a three-point flexural test method. The size of the specimen was 80 mm × 12.5 mm × 2 mm. And they were tested at the crosshead movement rate of 2 mm/min, with the span of 64 mm. At least five measurements were performed for each composite and get the average values of flexural strength and modulus.

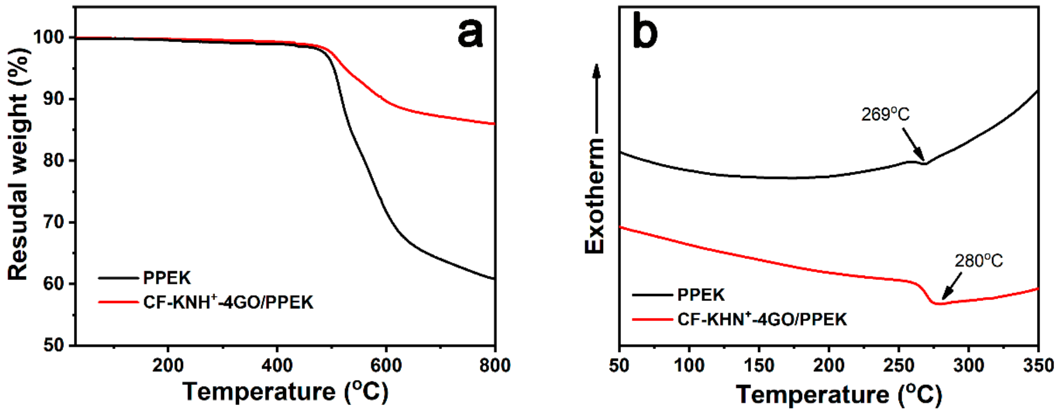

Figure S3. (a) TGA Spectra of PPEK and CF-KHN$^+$-4GO/PPEK; (b) DSC Spectra of PPEK and CF-KHN$^+$-4GO/PPEK;.

The thermal stability and T_g of PPEK and CF-KHN$^+$-4GO/PPEK composites was characterized TGA and DSC, and the thermograms were shown in Fig.S3. PPEK is a kind of high-performance special engineering plastics containing phthalazinoe in the main chain, which exhibits excellent thermal stability (the temperature at weight loss 5% is 503°C) due to the twisted non-coplanar structure in PPEK strengthen molecular chain entanglement and destroy the regularity of the molecular chain. The more excellent thermal stability is also observed for the CF-KHN$^+$-4GO/PPEK composites, and

temperature at weight loss 5% ($T_{d5\%}$) is enhanced by 22°C. As the carbon fiber scale effect restrict the movement of polymer chains, T_g of CF-KHN^{+}-4GO/PPEK composites (280°C) improve 11°C compare with PPEK.
